# The Influence of Gifted Children’s Stress Management on Parental Stress Levels

**DOI:** 10.3390/children11050538

**Published:** 2024-04-30

**Authors:** Maria Assunta Zanetti, Francesca Sangiuliano Intra, Livia Taverna, Antonella Brighi, Carlo Marinoni

**Affiliations:** 1 Department of Brain and Behavioral Sciences, University of Pavia, 27100 Pavia, Italy; zanetti@unipv.it (M.A.Z.); carlo.marinoni01@universitadipavia.it (C.M.); 2 Faculty of Education, Free University of Bolzano-Bozen, 39042 Bressanone-Brixen, Italy; livia.taverna@unibz.it (L.T.); antonella.brighi@unibz.it (A.B.)

**Keywords:** gifted children, emotional intelligence, emotional adjustment, parent–child relations, family relations

## Abstract

This study explores the associations between gifted children’s stress management and parental stress level. A sample of 78 primary school children and their 76 parents took part in this study. Children were screened for intelligence and emotional quotients, while parents were tested for stress levels. Results show that the more children are aware of their stress-management skills, the less parents are stressed out. Moreover, the intelligence quotient is not significant in mediating this association, supporting the idea that it is not an a priori protective factor from a developmental perspective. The study findings suggest that when a child is equipped with the skills to handle stress by harnessing their emotional intelligence, it can have a beneficial effect on the entire family’s well-being. Given that these skills can be developed, and the significant positive influence they have on a child’s growth and adaptation, it is essential to offer specialized educational programs to gifted children. These programs should aim to enhance their emotional skills, which, in turn, can indirectly bolster the psychological health of the family unit as a whole.

## 1. Introduction

### 1.1. Giftedness’ Definition

The majority of researchers identify giftedness in those individuals who have the potential to demonstrate exceptional abilities in one or more of the following areas, with respect to one’s peers: general intellectual abilities, specific aptitudes, or creative thinking [1]; however, it is still difficult to provide a unique definition of giftedness accepted by the entire scientific community [1]. The National Association for Gifted Children (NAGC) defines gifted students as those who possess the ability to perform at higher levels than their peers of similar age, background, and exposure in one or more areas. Gifted students are a heterogeneous group, comprising individuals from different racial and ethnic backgrounds, cultures, and socio-economic statuses. They require access to appropriate learning opportunities to reach their full potential. In this perspective, it is of utmost importance to consider that some of these students may have learning or processing disorders, necessitating specialized intervention and accommodation. Accordingly, tailored educational experiences are required to meet the needs of gifted students to meet their changing needs [2].

According to Newman’s definition from 2008, being gifted is typically associated with an IQ score of 130 or higher. However, Ruf and Valley suggest in their study [3] that this threshold could be lowered to 120. Children who possess high cognitive potential may display precocity in language development, possess exceptional abstract-reasoning skills, exhibit extreme curiosity, and have an outstanding memory; however, they are stigmatized, and their potential is often associated with success in an individual’s education and life trajectory [4]. However, giftedness is a complex concept that encompasses various components and cannot be limited to the mere IQ score. Studies in the scientific literature suggest that giftedness consists of high academic performance, high intellectual ability, social intelligence, task commitment, and creativity, making the IQ just one of the giftedness components [5,6,7]. Accordingly, the Differentiated Model of Giftedness and Talent [8] involves six components, among which are intrapersonal catalysts and the chance factor, highlighting the complexity of giftedness and talent. Moreover, co-cognitive traits, social capital, and family–school factors have been included, emphasizing the importance of personal and environmental attributes in nurturing giftedness [9]. Finally, original problem-solving strategies and creativity have been considered as crucial components of giftedness [10], showing the different nature of gifted individuals and the multifaceted aspects that define their exceptional abilities.

Such a plethora of personal and environmental dimensions support the idea that characteristics associated with giftedness, such as high sensitivity, intensity, and intellectual, emotional, and imaginative excitability may constitute risk factors for the development of emotional–behavioral problems [11,12,13].

Indeed, they may face challenges in identifying, managing, and regulating their emotions, as well as in developing social-relational skills, exhibiting an extreme sense of perfectionism and high cognitive activation, which could cause behavioral-management issues [14]. Accordingly, gifted children present an asynchronous development of cognitive, socio-emotional, and psychomotor abilities [15], and feelings of inadequacy and misunderstanding due to society’s expectations [16].

During the past 40 years, Italy has invested human and economic resources in developing programs, tools, and teacher training to meet the educational and emotional needs of students with learning disabilities, neglecting the educational needs of students of uncommon ability, high IQ, and creative potential. Italian society’s perception of high-ability students is that they are already a privileged group who will do quite well without special services. Compared to other European countries, Italy has been slower to respond to the educational needs of high-ability students who are under-challenged in schools due to a lack of awareness of their too-long-ignored educational needs. Although there is no specific legislation and no national educational measures have been adopted, actual school regulations make clear reference to the need for promoting the development of students’ potential and talents. In 2015, the law n. 107, called “*Buona Scuola*”, set the grounds for a review of current educational teaching strategies to support talented students through “Educational Flexibility”, referencing to the use of flexible instruments. More recently, in 2019, a specific note N. 562 “Clarifications for students with special educational needs” also provides for the possibility for gifted students to adopt specific teaching methodologies, such as compaction, differentiation, acceleration, and enrichment at both individual and class levels from an inclusive perspective. 

### 1.2. Giftedness and Emotional Intelligence 

The importance of emotional intelligence (EI) in the education and development of gifted individuals is widely recognized. EI has a significant impact on the social and emotional well-being of gifted individuals and influences how gifted children interact with their external environment, representing a critical factor in shaping their overall development [17]. The scientific literature [18] provides different definitions of EI, while the Encyclopaedia of Applied Psychology [19] identifies three major conceptual models: the Salovey–Mayer model [20], the Goleman model [21], and the Bar-On model [22]. This study specifically focuses on the Bar-On model, as the youth version of the Emotional Quotient Inventory developed by Bar-On and Parker [23] has been employed to investigate EI. 

The Bar-On proposal suggests that emotional and social intelligence encompasses a set of interrelated emotional and social skills that empower individuals to comprehend their own and others’ emotions, build meaningful relationships, and navigate the social challenges of everyday life. These skills involve being able to identify and understand one’s own mental states (emotions, beliefs, thoughts), as well as being able to understand and engage with the emotional states of others. It also includes the ability to regulate and manage emotions effectively, adapt to change, address personal and interpersonal challenges, and use positive emotions to motivate oneself in achieving personal goals and reaching one’s full potential [24]. Accordingly, physical, cognitive, social, and emotional developments present a relationship of interdependence and constant interaction [25]. Investigating gifted children’s EI allows us to understand how they perceive themselves, investigating their functioning and psychological wellbeing beyond their IQ score. 

Individuals with higher intellectual capacity often experience a mismatch between their mental and emotional growth, unlike individuals with average intellectual abilities. This kind of asynchronous development may manifest in several ways, such as advanced intellectual skills coupled with social or emotional immaturity, or a strong inclination towards specific interests while neglecting others. Due to their exceptional abilities, gifted individuals are more vulnerable and require specialized parenting, teaching, and counseling to reach their full potential [26].

Due to their asynchronous development, children may develop psychosocial problems, e.g., socially inappropriate behavior, difficulties in relating to peers, and inability to cope with stress [27,28], persisting into adulthood [29]. 

Although giftedness has been the subject of substantial research, an exhaustive comprehension of this phenomenon requires delving into both its cognitive and social-emotional facets [30]. Nevertheless, most studies have primarily addressed cognitive factors, leading to a restricted understanding of the complete spectrum of giftedness [31]. Within the scientific literature on gifted children, there is an ongoing debate regarding their emotional adaptability and the association between EI and giftedness [32,33,34]. Although other studies have deepened the differences in EI between gifted and non-gifted individuals, highlighting that gifted individuals tend to score higher on the adaptability and intrapersonal subscales of emotional intelligence and in line with the standard on the stress management and interpersonal skills subscales [35,36,37,38,39], gifted children exhibit greater resiliency to stress despite the fact that they also tend to experience such an emotion more intensely and require additional support to alleviate it effectively [40]. 

### 1.3. Gifted Children’s Stress Management

According to Moon [28], gifted children require support in addressing psychosocial issues that arise from social isolation and peer rejection as the primary result of the inability of social and educational environments to manage to their needs rather than their exceptional abilities themselves [28,41]. 

Moreover, other reasons underlying gifted children’s high levels of stress are related to their perfectionist tendencies, heightened sensitivity, social challenges, external pressures [42], over-demanding adults, and undemanding schoolwork [43,44,45]. 

Sustained and high levels of stress can have detrimental impacts on an individual’s mental and physical health. Among the most severe outcomes is burnout, a state of complete exhaustion that can result in a profound sense of depletion and fatigue. Burnout may also precipitate social withdrawal, as individuals may choose to isolate themselves from others due to the overwhelming feelings of stress and exhaustion [44]. Moreover, the issue of high stress levels in gifted children is compounded by the pressure exerted by adults to excel in academic performance and achieve exceptional results [46], creating an additional negative impact and a need to effectively manage the intense competition that arises in such situations [47]. 

In this regard, the role of adults, specifically parents, plays a key role in emotional development and in having a good quality of life, considering that they impact the development of their children’s emotional competencies, especially concerning the stress management skills [48,49,50]. The impact of having a child with unique abilities and needs is not limited to the individual child but extends to the entire family unit. This results in a bidirectional relationship between the child’s needs and the overall wellbeing of the family in which parents may experience stress due to this reciprocal relationship [51]. 

### 1.4. Stress Management for Gifted Children’s Parents

Although the role of parents is crucial, the scientific literature has under-investigated the causes of stress for gifted children’s parents and their parenting experiences [52,53,54,55,56]; making their children feel warmth and emotionally supported affects their development [57].

Parental stress is a term used to describe the emotional and cognitive reactions of parents to the various challenges that arise from the complex and multifaceted responsibilities of parenting [58], particularly in the context of parenting gifted children, where challenges can result in a range of psychological and physiological responses [52,53,54,58,59]. 

Specifically, the challenges faced by parents of gifted children are distinct and often result in the children experiencing elevated levels of stress [53,54,58,59] that are primarily linked to the unique educational requirements of gifted children; their needs are frequently not met by conventional schooling systems and they perceive a lack of understanding and support from society as a whole [60,61,62]. 

Morawska and Sanders [52] pointed out that parents require support not only to manage the school-educational needs but also to manage the difficulties associated with parenting, considering that family resilience promotes positive outcomes in managing and avoiding the stress of parenting gifted children and supporting their coping strategies [63,64,65,66]. 

Exploring parental stress is a crucial aspect as it has a direct impact on the parenting quality and the parent–child relationship [67,68]. Parents of gifted children may encounter a sense of incompetence and distress that could lead to unsuitable responses to the child’s adverse emotional state. These responses are likely to be linked with the emergence of negative emotional traits and low emotional competency [69] that eventually results in different perceptions about stress with a significant discrepancy between parent and children [32]. 

Parental stress can stem from a perceived lack of competence and control in meeting their children’s demands and aspirations. This can manifest in a variety of emotional reactions and actions, including but not limited to anxiety, exasperation, and even aggression, which can adversely affect the mental and physical health of both parents and children. Understanding the root causes of parental stress is an important step in developing effective strategies for managing and reducing its negative effects.

Undeniably, giftedness is a critical factor that can have a significant impact on family dynamics, which, in turn, plays a crucial role in shaping and nurturing giftedness. Therefore, it is imperative to acknowledge the interplay between giftedness and family and comprehend how they can mutually influence each other [70].

It must be emphasized that the characteristics of gifted people, including perfectionism and asynchrony, make parenting difficult [55]. Indeed, stress-management skills are crucial for enhancing child–parent relationships and overall wellbeing. The relationship between stress response, stress-management techniques, and subjective wellbeing highlights the importance of integrated stress-management approaches for promoting overall wellbeing [71].

### 1.5. Current Study

In this study, we aim to explore whether the ability of gifted children to cope with stress is associated with a perceived reduction in stress by parents. Moreover, we aim to deepen the role of the IQ in order to understand whether it is involved in such a dynamic. The ultimate objective is to identify insights that can inform the development of educational strategies to promote family wellbeing and facilitate adaptive child development. In light of the theoretical framework presented, the research questions are:

1. Is the gifted child’s ability to cope with stress a predictor of a reduced parental stress level? Specifically, we hypothesized that gifted children with superior stress-management skills can help in reducing parental stress [72]. Indeed, research has indicated that parents who are dealing with their own emotional challenges may have fewer coping resources and may experience heightened stress due to their children’s challenging behaviors [73]. Moreover, it has also been shown that child outcomes tend to be poorer when caregivers are highly distressed or are facing negative mental health outcomes themselves [74].
2. Is IQ a significant mediator in the relationship between the child’s stress management and the parent’s perceived stress level? In the literature, EI has been shown to be a crucial element in stress management [75]; however, little is known about the role of IQ in the link between EI and stress management [76].

## 2. Materials and Methods

### 2.1. Participants and Procedure

This study involves 78 gifted children (Male = 64; Female = 14; IQ mean = 135; SD = 8.39) aged between 7 and 10 (M = 8.77; SD = 0.78). The sample was geographically heterogeneous, with subjects from all over Italy. All children were Italian native speakers and attended primary school without neurological conditions or emotional/behavioral problems. 

The parents sample was composed of 76 parents (Male = 76, Female = 76), age range was 33–50 years old for females (M = 42.4; SD = 4) and 35–66 years old for male (M = 45.8; SD = 6.04). The level of education expressed in years of formal school was 17.75 for males and 18.2 for females.

Data collection was performed at the LabTalento, a research laboratory that belongs to the University of Pavia (Italy). Children and adolescents came from the all the Italian regions and were assessed by a team of experts with respect to cognitive, social, and emotional dimensions. These data were collected along with a complete IQ assessment and were analyzed for research purposes. The inclusion criteria required children to have an IQ score of 120 or higher, and both parents had to participate in the research. Children who scored below 120 on the IQ assessment and those identified as having double exceptionality were excluded due to the small sample size, which prevented thorough exploration of dynamics within such subgroups. The designated ethics committee at the University of Pavia approved the use of the data for research purposes (protocol number 158/23). All parents provided informed consent for both themselves and their children. The children were also informed about the procedures and the purpose of the research before participating in the assessment.

Before engaging in emotional intelligence assessments, all children underwent an initial evaluation of their Intelligence Quotient (IQ) using standardized and age-appropriate measures. The assessment was conducted individually by trained psychologists to ensure accuracy and reliability of the results. The assessment was conducted in a dedicated room specifically designed for psychological assessments. This room was soundproofed to minimize external noise and distractions; responses are recorded by the administering psychologist.

### 2.2. Measures

The children’s emotional intelligence assessment consisted of the youth version of the EQ-I:YV—Emotional Intelligence Quotient Inventory [23] that is a questionnaire for assessing emotional intelligence in children and adolescents aged 7 to 18. It is a self-administered tool based on Bar-On’s model of emotional and social intelligence [77]; this instrument represents the youth version of the Emotional Quotient Inventory (EQ-i), widely used to measure emotional intelligence in adults [77]. According to Bar-On’s model, emotional intelligence encompasses emotional, personal, and social dimensions, involving understanding oneself and others, adapting to environmental changes, and managing emotions. The EQ-I:YV has been validated on an Italian sample and all the scores are calculated with reference to the published normative sample [77]. It is composed of 60 items divided into five latent dimension: (1) Intrapersonal, which assesses the capacity to comprehend the emotions of others and to communicate about that within the relational interactions; (2) Interpersonal, which refers to one’s ability to have fair and satisfactory interpersonal relationships and to understand, consider, or appreciate the emotions of others; (3) Stress management, which is the individual’s ability to manage and control emotions, and respond calmly to stress; (4) Adaptability, referring to the ability to solve problems and manage changes effectively, being flexible and realistic; (5) General Humor, which represents the effect of socio-emotional functioning. Furthermore, the questionnaire incorporates an additional latent dimension known as “positive impression” to ensure the reliability of the responses in term of social desirability.

Each item must be rated by using a 4-point Likert scale, where 1 means completely disagree and 4 means completely agree.

The entire assessment procedure, including the IQ assessment, spanned over two days, during which there were scheduled breaks to avoid excessive fatigue for the children. They were free to stop the evaluation at any time at their own choice.

The Parenting stress Index [78] is a widely used tool designed to assess the stress experienced by parents involved in the parent–child system.

The questionnaire contains 36 items that require participants to rate each item using a 5-point Likert scale. A rating of 1 indicates strong disagreement, while a rating of 5 indicates strong agreement. Some items have non-Likert response options that delve into how parents feel about being good parents or how challenging/easy it is to perform the task mentioned in the item. In the present study, we use the Italian version validated by Guarino and colleagues 2008 [79] and distributed by Giunti. This questionnaire is designed to delve into the various challenges parents face in their daily responsibilities of caregiving, such as feeding, bathing, and managing household routines. It also examines how parents perceive their child’s behavior and if it contributes to their stress levels. The quality of the parent–child relationship is evaluated, along with questions about the availability of social support from family, friends, or community resources. In addition, the questionnaire assesses the parent’s confidence in their ability to provide effective care for their child and considers any recent life events or stressors that may impact their overall stress levels. These comprehensive inquiries provide a detailed understanding of parental stressors and their impact on caregivers’ scoring.

## 3. Results

Analyses were carried out using Jamovi Version 2.3 (The Jamovi project, 2022; R core Team).

Data were tested for normality using the Shapiro–Wilk test: Z_Children_IQ = 0.969, *p* = 0.060; Z_Stress_Management = 0.976, *p* = 0.157; Z_Parental_Stress_Total_index = 0.977, *p* = 191; all variables were near normal distribution and for this reason we conducted correlation analysis using Pearson’s rho.

Descriptive statistics about Emotional Intelligence Quotient Inventory (Eqi) are reported in Table 1. The Eqi subscales include Positive Impression (α = 0.527; ω = 0.572), General Humor (α = 0.704; ω = 0.727), Adaptability (α = 0.683; ω = 0.707), Stress Management (α = 0.634; ω = 0.648), Interpersonal (α = 0.626; ω = 0.653), and Intrapersonal (α = 0.567; ω = 0.592). The dataset comprises 74 to 76 observations, with the presence of missing data varying across the subscales, with the highest number of missing data points of 2. Concerning measures of central tendency, we find the mean scores for the subscales, which offer insights into the average performance or level within each domain, spanning from 91.0 to 107, showing the variation in emotional intelligence across these dimensions. Median scores closely align with the mean scores, indicating relatively balanced distributions within each subscale. Regarding variability, the range observed was from 12.3 to 15.7, providing a sense of the degree of variation within each dimension. Moreover, each subscale’s minimum and maximum scores offer insights into the range of observed values. The minimum score range, excluding the subscale of positive impression as a control scale, was 77 in the adaptability subscale, while the general humor and interpersonal subscales reached the maximum scores of 130. Finally, all the participants scored above the threshold on the inconsistency index and none were excluded.

For the PSI-Female group, the dataset comprises 76 respondents. There is no missing Parental Distress (PD; α = 0.749; ω = 0.770), Parent–Child Dysfunctional Interaction (P-CDI; α = 0.839; ω = 0.847), Difficult Child (DC; α = 0.858; ω = 0.869), and Toral Score (TS) are 58.9, 53.9, 56.3, and 62.4, respectively, reflecting the average stress levels within each sub-component for mothers. The median values for these sub-components are 60.0, 50.0, 55.0, and 65.0, while the standard deviations are 25.4, 25.7, 26.8, and 26.5, indicating the degree of variability in the scores around the means for the PSI-Male group, a paired sample of 76 respondents is observed, with no missing data. The mean scores for DR, PD (α = 0.815; ω = 0.839), P-CDI (α = 0.786; ω = 0.815), DC (α = 0.860; ω = 0.863) and TS in this group are 54.2, 51.7, 57.3, and 57.0, respectively, indicating the average stress data in any of the sub-components. The mean scores for Defensive Response (DR), levels experienced by fathers in each sub-component. The median values for these sub-components are 50.0, 50.0, 50.0, and 60.0, while the standard deviations vary between 25.2, 25.1, 52.6, and 26.5, reflecting the degree of dispersion in the scores. In the female sample, all the participants scored above the threshold (<=10), while in the male sample, one participant scored below the threshold in the Defensive Response component that is a control subscale to assess the reliability of the participants’ responses and for this reason that participant’s responses have been excluded from further analyses. Descriptive statistics are provided in Table 2.

To explore the potential link between parents’ stress perception and their children’s stress coping abilities, we developed a comprehensive parental stress index. This index was calculated by averaging the total stress scores of both mothers and fathers. Employing a total stress index offers a comprehensive and inclusive approach, integrating the perspectives of both mothers and fathers to provide a wider understanding of parental stress within the family dynamic. By aggregating stress scores from both parents, it captures a broader spectrum of parental experiences, enhancing the robustness and reliability of findings. This measure facilitates easier comparison and interpretation of results, simplifying data analysis and enabling clearer conclusions regarding the association between parental stress and children’s coping abilities (PSI_total_mean_ = 59.7; SD PSI_total = 22).

Considering the strong negative correlation between PSI_total and CSM (roh = −0.333, *p* < 0.01), we computed a linear regression model to verify whether the CSM could be consider as a predictor of the level of parental stress with the eventual covariation role of the IQ. Results confirm the hypothesis, although the IQ was not significant as a mediator. Data are provided in Table 3.

## 4. Discussion

The present study aims to investigate whether and how the stress experienced by parents of gifted children is associated with children’s stress-management skills, to improve the understanding of this dynamic to foster families’ wellbeing and promote gifted children’s adaptive development. Moreover, the study explores the impact of children’s emotional intelligence and QI on this connection.

Our results show that the less competent the child feels in handling stress, the higher the parent’s stress levels. This finding is in line with literature, as there is a two-way relationship of mutual influence between parents and children [49]. Research has shown that there is a transactional relationship between parenting stress and child behavior problems, indicating that higher levels of stress in parents can be influenced by the behavior of the child [80] and our findings support the reverse dynamic, highlighting how a child’s enhanced competence can mitigate parental stress. From this perspective, a child’s emotional competence emerges as a protective factor not only for their own wellbeing but also for the harmony within family relationships.

As it is evident in the literature, being a parent is a difficult task, especially when it comes to gifted children, who have different needs than non-gifted peers, increasing parental stress and anxiety regarding the future development and upbringing of gifted children [53,55,81].

The outcomes of the present study can be attributed to the nature of interdependence between parents and children, as parents frequently struggle to provide adequate and timely responses to their children’s needs starting from an early age. Indeed, competent and emotionally regulated parents can positively influence their child’s self-esteem and foster the development of high functional emotional intelligence. This includes managing emotion regulation and stress effectively, making them more resilient in facing challenges as a family system [50]. Moreover, authoritarian parenting may detrimentally impact children’s psychological wellbeing by creating an unwelcoming environment, undermining their self-esteem, and impeding emotional regulation [82,83,84].

Another challenge arises when parents of gifted children also have non-gifted children and employ the same parenting approaches uniformly across all their children. This approach may overlook the unique needs and characteristics of each child, leading to behaviors that are not universally effective or appropriate [55].

While gifted children go through the same developmental stages as their non-gifted peers, their experience is qualitatively different. Indeed, gifted children have unique educational needs that may differ from those of non-gifted children. Due to their advanced metacognitive thinking skills and distinct learning strategies, gifted children often require specialized academic support that extends beyond high IQ levels [85].

Indeed, it has been suggested that metacognitive knowledge and control tend to develop earlier in gifted students compared to non-gifted students [86].

Interestingly, our results show that the IQ is not significantly involved in the relationships between children stress-management skills and parental stress, supporting the idea that emotional intelligence is an independent factor involved in children’s wellbeing and, for this reason, it should be nurtured by educational institutions [87]. Accordingly, while cognitive ability or IQ may have a positive correlation with creativity and divergent thinking, it seems to be negatively associated with emotional intelligence [88], suggesting, in line with our findings, that high IQ does not determine emotional abilities in gifted children. Moreover, the overemphasis on a child’s giftedness, solely based on IQ scores, may limit the development of a more comprehensive parent–child relationship, and potentially hinder the child’s social and emotional adjustment [55]. Therefore, it is essential to consider emotional intelligence alongside cognitive abilities when identifying and supporting gifted children. By recognizing and fostering their emotional intelligence, educators and parents can better cater to the holistic needs of gifted children, ensuring their emotional wellbeing aligns with their intellectual capabilities.

In this perspective, research conducted by Rinn and Majority [89] highlighted the phenomenon of asynchronous development, where children’s advanced cognitive abilities and intense emotions create unique inner experiences that differ from the typical development. The extent of asynchrony in an individual’s cognitive and emotional development is directly proportional to their intellectual capacity. Accordingly, Peterson [32] highlights that gifted children require distinct attention and education to help them reach their full potential. In the absence of suitable academic guidance, gifted children may lose motivation for education, display suboptimal academic performance, or even abandon their schooling altogether. Moreover, ensuring that gifted children have access to specific education is a matter of equity and inclusion, in line with the sustainable goal proposed by the UNESCO Agenda 2030 (UNESCO, 2017). Regardless of their abilities, all children should have the opportunity to reach their full potential: neglecting the needs of gifted children can perpetuate educational disparities in contrast with sustainable goals of reaching quality education and reducing inequalities (UNESCO, 2017). These experiences often make children feel inadequate, lonely, and excluded, experiencing negative emotions that impact and influence parental emotions and ability to manage stress [90].

Difficulties may arise because, given parents’ lack of knowledge of the gifted world and their lack of preparation, they are very often unaware of the specific causes of their children’s stress due to academic commitments. This perceived helplessness of parents causes stress, which in turn affects their children’s wellbeing [32]. On the other hand, overemphasizing a child’s giftedness can potentially limit the development of a broader parent-child relationship, affecting the child’s social and emotional adjustment [91]. For this reason, it is crucial to create support groups also among parents in which they can educate themselves about giftedness and seek mentorship to meet their children’s educational needs [92]. Hence, effective cooperation between families and schools is essential for creating conducive environments that facilitate the successful development of gifted students [93]. In this framework, developing successful stress-management skills and cultivating emotional intelligence can have a positive impact on family and social relationships, but its also act as a protective factor against the risk of school drop-out. By learning how to manage stress and regulate emotions, gifted children become better equipped to navigate challenges and setbacks, reducing the likelihood of academic struggles and increasing the overall psychological wellbeing.

Despite the presence of studies e.g., [94] that exclude the impact of EI on school success, more recent research [95] showed that a high EI, with relatively higher stress management, corresponded to greater school success. Chan [96] found that gifted children faced strong academic pressure, so stress-management skills allowed them to manage academic expectations.

The existing literature on the impact of gifted children on their parents’ psychological wellbeing is limited. Therefore, this study contributes by providing relevant data to support existing research, improving the understanding of those mechanisms underlying gifted children and respective families’ wellbeing along with the perspective of adaptive development. It appears that both parents and gifted children experience stress due to insufficient resources, such as educational and social support, to help them manage their daily challenges. This mutual influence underscores the need for better solutions to support both gifted students and their parents in coping with the demands of life.

These findings highlight the concerning and intricate nature of the situation. They highlight the inadequacy of available resources within the school system to support parents in addressing the unique needs of gifted children. There is a pressing need for teacher training programs aimed at enhancing understanding of the distinct characteristics of gifted children, whose potential is frequently misperceived as a challenge and subsequently mismanaged. Without proper recognition and support, gifted individuals may resort to concealing their abilities to fit in with peers or, conversely, exhibit frustration through hyperactive or disruptive behavior. Addressing these issues is paramount to fostering an environment conducive to the holistic development of gifted youth. On the other hand, the parents of gifted children and the gifted children themselves feel alone, without any kind of support and help [53,81,82,97], which increases the uncertainty and stress of not having the right tools to deal with such a situation.

From an academic point of view, the need for interventions for gifted children stems from the need to compensate for the difficulties and stress they may experience [98]. Having a lack of interests in common with non-gifted peers, feeling different, or being perceived as a know-it-all can lead to social isolation and perceived stress [28,41,42,43,44,45,46,47,48,49,50,51,52,53,54,58,59,60,61,62,63,64,65,66,67,68,69,70,72,75,76,78,79,89,90,94,95,96,99,100].

From a parenting point of view, the scientific literature emphasizes that parenting intervention for parents of gifted children is under-researched [52].

Intervention programs to support gifted parents (e.g., triple P: Positive Parenting Program) have been found to be fruitful in effectively supporting child development, including managing emotions, stress, and problematic behavior and support parents in their educational role, enabling them to cope with their children’s developmental needs [52].

Parental stress may be present because parents very often experience their children’s giftedness not as a resource, but as a pathology. Perhaps this parental perception may stem from the lack of social and school support, which makes giftedness appear as something unknown and difficult to deal with, causing stress and an inability to cope. Educational institutions could provide parental support at the start of the school year to assist both parents and teachers in addressing the unique needs of gifted children. By doing so, gifted children and their parents could feel supported and understood, rather than isolated and disconnected from their community. This approach has the potential to create a more inclusive and empowering educational environment for all students involved.

The significance of these findings should make us reflect on the importance of the giftedness issue and the need to adopt appropriate strategies that support gifted parents and gifted children as children and learners. It is crucial since the difficulties of the gifted do not only concern one educational level; giftedness can be viewed as a lifelong developmental challenge, as the nature of cognitive and socio-emotional growth is dynamic and malleable [101].

## 5. Conclusions

In conclusion, this study delves into the complex dynamics of stress experienced by parents of gifted children, seeking to uncover the interplay between parental stress levels and children’s stress-management skills based on the hypothesis that a more emotionally competent child causes fewer concerns in parents, who consequently are less stressed. By exploring the influence of emotional intelligence and IQ on this relationship, we have gained valuable insights into the wellbeing mechanisms underlying the relationships within gifted families. Our findings underscore the bidirectional nature of the parent–child relationship, where parental stress, proven in the scientific literature to be a predictor of the emotional extent of children, can itself be influenced by children’s abilities to manage stress effectively. This highlights the importance of nurturing emotional competence in children, not only for their own wellbeing but also for promoting harmony within family dynamics. Furthermore, our results emphasize the distinct educational needs of gifted children, extending beyond mere cognitive abilities to encompass emotional intelligence and metacognitive processes. While parental support and interventions are crucial for fostering the development of gifted children, our study reveals a gap in research on parenting interventions specifically tailored to the needs of gifted families in terms of children’s stress-management strategies. Finally, our findings underscore the need for collaborative efforts between educational institutions, parents, and communities to create inclusive environments that support the unique needs of gifted children and their families. By recognizing and addressing these needs, we can empower gifted individuals to reach their full potential and contribute meaningfully to society.

This study also presents some limitations. Indeed, it is recommended that future studies take a longitudinal approach in order to improve the understanding of the sequence of events over time and deepen the causal relationships among variables. Cross-sectional data cannot capture the dynamic nature of behavior, attitudes, or experiences over time. Additionally, in our study, the regression model only explains a limited variance portion; therefore, future studies are necessary to further strengthen this insight. Another aspect that should increase the comprehension of this dynamic is assessing the parents’ IQ to explore whether the giftedness is shared between parents and child. Recent research has shed light on the multifaceted nature of intellectual giftedness, revealing the intertwined contributions of genetic and environmental factors. While genetic predispositions provide a foundation for giftedness, environmental influences such as educational opportunities, socio-economic background, and family support also significantly shape cognitive development. Understanding the complex interplay between these factors is essential for unveiling the mechanisms that are giftedness-related and designing effective interventions to nurture the potential of gifted individuals [102].

Moreover, due to the reduced sample size, accounting for specific circumstances, such as single-parent families or twice-exceptional children was not feasible; future studies should consider examining such sub-samples in order to provide a more representative result. This is because the challenges faced by these children are not only related to a lack of resources to cope with their giftedness, but also due to difficulties in managing their weaknesses. These challenges can also lead to high levels of parental stress [84,103].

Finally, it would be valuable to investigate the influence of teachers in future research to gain an objective understanding of children’s abilities and difficulties. Analyzing teachers’ perspectives can also enhance the understanding of their perceptions of giftedness as well as their roles as school caregivers. This, in turn, may help create effective educational interventions to support children and families.

## Figures and Tables

**Table 1:** Descriptive statistics of the children EQ-i subscales.

	Eqi—Children					
	Positive Impression	General Humor	Adaptability	Stress Management	Inter.	Intra.
N	74	76	76	75	76	76
Missing	2	0	0	1	0	0
Mean	97.8	98.8	107	91.0	103	94.4
Median	99.0	99.5	108	92.0	105	93.0
Standard deviation	15.7	15.3	12.3	15.2	15.1	15.5
Minimum	60	66	77	60.0	66	69
Maximum	130	130	129	122	130	128

**Table 2:** Descriptive statistics of the Parental Stress Index (PSI) sub-component, specifically Defensive Response (DR), Parental Distress (PD), Parent–Child dysfunctional interaction (P–CDI) and Total Stress (TS). Data are provided for both mother and father.

	**PSI—Female**				
	**DR**	**PD**	**P-CDI**	**DC**	**TS**
N	76	76	76	76	76
Missing	0	0	0	0	0
Mean	58.9	53.9	56.3	72.4	62.4
Median	60.0	50.0	55.0	85.0	65.0
Standard deviation	25.4	25.7	26.8	26.5	26.5
Minimum	15	10	10	10	10
Maximum	100	100	100	100	100
	**PSI—Male**				
	**DR**	**PD**	**P-CDI**	**DC**	**TS**
N	76	76	76	76	76
Missing	0	0	0	0	0
Mean	54.2	51.7	57.3	65.2	57.0
Median	50.0	50.0	50.0	67.5	60.0
Standard deviation	25.2	25.1	52.6	27.5	26.5
Minimum	5	1	5	10	5
Maximum	95	100	456	100	100

**Table 3:** Linear regression analysis results: model fit and coefficients for parental stress total index (PSI_total) as a dependent variable, children stress management (CSM) as a predictor and intelligence quotient (IQ) as a covariate.

			Overall Model Test					
Model	R	R^2^	F	df1	df2		* p *	
1	0.344	0.118	9.78	1	73		0.003	
Model Coefficients—PSI_total								
Predictor	Estimate	SE	t	* p *				
Intercept	104.471	14.683	7.12	<0 .001				
CSM	−0.497	0.159	−3.13	0.003				

Note. Weighted by ‘IQ’.

## Data Availability

Data is unavailable due to privacy restrictions.

## References

[1] Zanetti M.A. Una doppia difficoltà in classe: I bambini ad alto potenziale. Proceedings of the 12th Applied Behavior Analysis Conference.

[2] Coleman L.J., Cross T.L. (2021). Being Gifted in School: An Introduction to Development, Guidance, and Teaching.

[3] Ruf D.L., Valley G. (2003). Use of the SB5 in the assessment of high abilities. Assess. Serv. Bull..

[4] Achter J.A., Lubinski D., Benbow C.P. (1996). Multipotentiality among the intellectually gifted: “It was never there and already it’s vanishing”. J. Couns. Psychol..

[5] Mohamed A., Elhoweris H. (2022). Perceptions of preschool teachers of the characteristics of gifted learners in abu dhabi: A qualitative study. Front. Psychol..

[6] Lavrijsen J., Verschueren K. (2023). High cognitive ability and mental health: Findings from a large community sample of adolescents. J. Intell..

[7] Chowkase A. (2022). Three c’s conception of giftedness: A call for paradigm shift. Gift. Educ. Int..

[8] Gagné F. (2000). A differentiated model of giftedness and talent (DMGT). Syst. Models Dev. Programs Gift. Talent..

[9] Renzulli J. (2002). Expanding the conception of giftedness to include co-cognitive traits and to promote social capital. Phi Delta Kappan.

[10] Keleş T. (2022). A comparison of creative problem solving features of gifted and non-gifted high school students. Pegem J. Educ. Instr..

[11] Bailey C.L. (2011). An examination of the relationships between ego development, Dabrowski’s theory of positive disintegration, and the behavioral characteristics of gifted adolescents. Gift. Child Q..

[12] Shahzad S., Begume N. (2010). Level of Depression in Intellectually Gifted Secondary School Children. Gift. Talent. Int..

[13] Dai D.Y. (2020). Assessing and accessing high human potential: A brief history of giftedness and what it means to school psychologists. Psychol. Sch..

[14] Zanetti M.A. (2014). Bambini ad Alto Potenziale (Gifted Children): Impariamo a Riconoscerli. QI—Questioni e idee in Psicologia—Il Magazine Online di Hogrefe Editore, NUMERO 20. https://qi.hogrefe.it/rivista/bambini-ad-alto-potenziale-gifted-children-imparia/.

[15] Guénolé F., Louis J., Creveuil C., Baleyte J.M., Montlahuc C., Fourneret P., Revol O. (2013). Behavioral profiles of clinically referred children with intellectual giftedness. BioMed Res. Int..

[16] Preuss L.J., Dubow E.F. (2004). A comparison between intellectually gifted and typical problems. Pol. Psychol. Bull..

[17] Chen X., Cheng L.J. (2023). Emotional intelligence and creative self-efficacy among gifted children: Mediating effect of self-esteem and moderating effect of gender. J. Intell..

[18] Mayer J.D., Salovey P., Caruso D., Sternberg R.J. (2000). Models of emotional intelligence. Handbook of Intelligence.

[19] Spielberger C. (2004). Encyclopedia of Applied Psychology.

[20] Salovey P., Mayer J.D. (1990). Emotional intelligence. Imagin. Cogn. Personal..

[21] Goleman D. (1998). Working with Emotional Intelligence.

[22] Bar-On R. (1997). The Emotional Quotient Inventory (EQ-i): Technical Manual.

[23] Bar-On R., Parker J. (2000). Emotional Quotient Inventory: Youth Version.

[24] Bar-On R. (2007). The impact of emotional intelligence on giftedness. Gift. Educ. Int..

[25] Diamond A. (2007). Interrelated and interdependent. Dev. Sci..

[26] Cross T.L., Anderson L., Mammadov S., Cross J.R., Roberts J.L., Inman T.F., Robins J.H. (2021). Social and emotional development of students with gifts and talents. Introduction to Gifted Education.

[27] Colangelo N., Davis G. (2002). Handbook of Gifted and Talented Education.

[28] Moon S., Neihart M., Reis S.M., Robinson N.M. (2002). Counseling needs strategies. The Social and Emotional Development of Gifted Children: What Do We Know?.

[29] Fiedler E.D. (1993). Square pegs in round holes: Gifted kids who don’t fit in. Underst. Our Gift..

[30] Ogurlu U. (2021). A meta-analytic review of emotional intelligence in gifted individuals: A multilevel analysis. Personal. Individ. Differ..

[31] Zeidner M. (2017). Tentative guidelines for the development of an ability-based emotional intelligence intervention program for gifted students. High Abil. Stud..

[32] Peterson J.S. (2009). Myth 17: Gifted and talented individuals do not have unique social and emotional needs. Gift. Child Q..

[33] Vialle W., Heaven P.C.L., Ciarrochi J. (2007). On being gifted, but sad and misunderstood: Social, emotional, and academic outcomes of gifted students in the Wollongong Youth Study. Educ. Res. Eval..

[34] Saggino A., Balsamo M., Di Sano S., Picconi L., Romanelli R. (2013). Giftedness between psychometric intelligence and emotional intelligence. Ric. Psicol.

[35] Colangelo N., Kelly K.R., Schrepfer R.M. (1987). A comparison of gifted, general, and special learning needs students on academic and social self-concept. J. Couns. Dev..

[36] Lupu V. (2012). Emotional intelligence in gifted and nongifted high school. Bull. Sci..

[37] Al-Onizat S.H. (2012). The relationship between emotional intelligence and academic adaptation among gifted and non-gifted student. Int. J. Hum. Sci..

[38] Lee S., Olszewski-Kubilius P. (2006). The emotional intelligence, moral judgment, and leadership of academically gifted adolescents. J. Educ. Gift..

[39] Schwean V.L., Saklofske D.H., Widdifield-Konkin L., Parker JD A., Kloosterman P. (2006). Emotional intelligence and gifted children. E-J. Appl. Psychol..

[40] Haberlin S., O’Grady P. (2018). Gifted from the “Inside out”: Teaching mindfulness to high-ability children. Gift. Educ. Int..

[41] Mofield E.L., Parker Peters M. (2022). Understanding the Interplay of Psychosocial Competencies in Talent Development: Typologies and Differences for Gifted Students. Roeper Rev..

[42] Haberlin S. (2015). Don’t stress: What do we really know about teaching gifted children to cope with stress and anxiety?. Gift. Talent. Int..

[43] Cross J.R., Cross T.L. (2015). Counseling the gifted: Past, present, and future directions. J. Couns. Dev..

[44] Fakolade O.A., Archibong I.E. (2013). Stress and intelligence: Understanding and encouraging the exceptionally gifted and talented learners to cope with stress. Afr. J. Psychol. Stud. Soc. Issues.

[45] Hébert T.P., Furner J.M. (1997). Helping high ability students overcome math anxiety through bibliotherapy. J. Second. Gift. Educ..

[46] Metha A., McWhirter E.H. (1997). Suicide ideation, depression, and stressful life events among gifted adolescents. J. Educ. Gift..

[47] Udvari S.J., Schneider B.H. (2000). Competition and the adjustment of gifted children: A matter of motivation. Roeper Rev..

[48] Martin-Krumm C., Fenouillet F., Csillik A., Kern L., Besançon M., Heutte J., Diener E. (2018). Changes in emotions from childhood to young adulthood. Child Indic. Res..

[49] Zeidner M., Matthews G., Roberts R.D., MacCann C. (2003). Development of emotional intelligence: Towards a multi-level investment model. Hum. Dev..

[50] Ramírez-Lucas A., Ferrando M., Sainz A. (2015). Influyen los estilos parentales y la inteligencia emocional de los padres en el desarrollo emocional de sus hijos escolarizados en 2° ciclo de educación infantil?. Acción Psicológica.

[51] Hermann K.M., Lawrence C., Cross T.L., Cross J.R. (2012). Family relationships. Handbook for Counselors Serving Students with Gifts and Talents.

[52] Morawska A., Sanders M.R. (2009). Parenting gifted and talented children: Conceptual and empirical foundations. Gifted Child Q..

[53] Jolly J.L., Matthews M.S. (2012). A critique of the literature on parenting gifted learners. J. Educ. Gift..

[54] Ritchie S. (2013). Giftedness 101, L.K. Silverman. Springer Publishing Company, New York, NY (2013), ((pbk) xiv, 292 pp.), ISBN: 978-0-8261-0797-8. Intelligence.

[55] Papadopoulos D. (2021). Parenting the exceptional social-emotional needs of gifted and talented children: What do we know?. Children.

[56] Zanetti M.A., Trombetta T., Rollè L., Marinoni C. (2024). Family Functioning and Internalizing and Externalizing Problems in Gifted Children. Eur. J. Investig. Health Psychol. Educ..

[57] Suldo S.M., Shaunessy-Dedrick E., Ferron J., Dedrick R.F. (2018). Predictors of success among high school students in advanced placement and international baccalaureate programs. Gift. Child Q..

[58] Deater-Deckard K. (1998). Parenting Stress.

[59] Keirouz K.S. (1990). Concerns of parents of gifted children: A research review. Gift. Child Q..

[60] Alsop G. (1997). Coping or counseling: Families of intellectually gifted students. Roeper Rev..

[61] Webb J.T., Gore J.L., Amend E.R., DeVries A.R., Kim M. (2008). A parent’s guide to gifted children. Gift. Talent. Int..

[62] Pelosi M., Montuori S., Zanetti M.A. (2021). Emotion and state of mind during lockdown: Focus on giftedness. Maltrattamento E Abus. All’infanzia.

[63] Masten A.S., Monn A.R. (2015). Child and family resilience: A call for integrated science, practice, and professional training. Fam. Relat..

[64] Magnuson K.A., Duncan G.J. (2004). Parent-versus child-based intervention strategies for promoting children’s well-being. Family Investments in Children’s Potential.

[65] Pfeiffer S.I., Reddy L.A. (2001). Mental Health Prevention Programs for Children.

[66] Stone L.L., Mares S.H., Otten R., Engels R.C., Janssens J.M. (2016). The co-development of parenting stress and childhood internalizing and externalizing problems. J. Psychopathol. Behav. Assess..

[67] Abidin R.R. (1992). The determinants of parenting behavior. J. Clin. Child Psychol..

[68] Rodgers A.Y. (1998). Multiple sources of stress and parenting behavior. Child. Youth Serv. Rev..

[69] Eisenberg N., Cumberland A., Spinrad T.L. (1988). Parental socialization of emotion. Psychol. Inq..

[70] May K.M. (2000). Gifted children and their families. Fam. J..

[71] Hepburn S.J., Carroll A., McCuaig L. (2021). Promoting stress management and wellbeing for teachers, A pilot study. Front. Educ..

[72] Zeidner M., Shani-Zinovich I., Matthews G., Roberts R.D. (2005). Assessing emotional intelligence in gifted and nongifted high school students: Outcomes depend on the measure. Intelligence.

[73] Davis N., Carter A.S. (2008). Parenting stress in mothers and fathers of toddlers with autism spectrum disorders: Associations with child characteristics. J. Autism Dev. Disord..

[74] Russell B.S., Hutchison M., Tambling R.B., Tomkunas A.J., Horton A.L. (2020). Initial challenges of caregiving during COVID-19: Caregiver burden, mental health, and the parent–child relationship. Child Psychiatry Hum. Dev..

[75] Görgens-Ekermans G., Brand T. (2012). Emotional intelligence as a moderator in the stress–burnout relationship: A questionnaire study on nurses. J. Clin. Nurs..

[76] Jung Y.H., Shin N.Y., Yang J.H., Lee W.J., Lee D., Choi Y., Kang D.H. (2019). Relationship among stress, emotional intelligence, cognitive intelligence, and cytokines. Medicine.

[77] Bar-On R., Parker J. (2012). Emotional Quotient Inventory: Youth Version.

[78] Abidin R.R. (1995). Parenting Stress Index: Professional Manual.

[79] Guarino A., Di Blasio P., D’Alessio M., Camicasca E., Serantoni G. (2008). Parenting Stress Index: Manuale.

[80] Neece C., Green S., Baker B. (2012). Parenting stress and child behavior problems: A transactional relationship across time. Am. J. Intellect. Dev. Disabil..

[81] Papadopoulos D. (2020). Psychological framework for gifted children’s cognitive and socio-emotional development: A review of the research literature and implications. J. Educ. Gift. Young Sci..

[82] Yazdani S., Daryei G. (2016). Parenting styles and psychosocial adjustment of gifted and normal adolescents. Pac. Sci. Rev. B Humanit. Soc. Sci..

[83] Pilarinos V., Solomon C.R. (2017). Parenting styles and adjustment in gifted children. Gift. Child Q..

[84] Ronksley-Pavia M., Pendergast D. (2020). Countering the paradox of twice exceptional students: Counter-narratives of parenting children with both high ability and disability. Routledge Handbook of Counter-Narratives.

[85] Alelyani S. (2021). Special educational need of the gifted and talented students in saudi arabia: A review paper. Int. J. Educ. Res. Rev..

[86] Kontostavlou E.Z., Drigas A. (2021). How Metacognition Supports Giftedness in Leadership: A Review of Contemporary Literature. Int. J. Adv. Corp. Learn. (Ijac).

[87] Casino-García A., Llopis-Bueno M., Llinares-Insa L. (2021). Emotional intelligence profiles and self-esteem/self-concept: An analysis of relationships in gifted students. Int. J. Environ. Res. Public Health.

[88] Furnham A. (2016). The relationship between cognitive ability, emotional intelligence and creativity. Psychology.

[89] Rinn A.N., Majority K.L. (2018). The social and emotional world of the gifted. Handbook of Giftedness in Children: Psychoeducational Theory, Research, and Best Practices.

[90] Lennard A.C., Scott B.A., Johnson R.E. (2019). Turning frowns (and smiles) upside down: A multilevel examination of surface acting positive and negative emotions on well-being. J. Appl. Psychol..

[91] Cornell D. (1989). Child adjustment and parent use of the term “gifted”. Gift. Child Q..

[92] Manasawala S., Desai D. (2019). Meeting the educational needs of a gifted child: A parent’s narrative. Gift. Educ. Int..

[93] Gali G., Fakhrutdinova A., Grevtsova G., Gali I. (2019). The cooperation between family and school as an important aspect in the development of gifted children. Humanit. Soc. Sci. Rev..

[94] Woitaszewski S.A., Aalsma M.C. (2004). The contribution of emotional intelligence to the social and academic success of gifted adolescents as measured by the Multifactor Emotional Intelligence Scale–Adolescent version. Roeper Rev..

[95] Parker J.D., Saklofske D.H., Keefer K.V. (2017). Giftedness and academic success in college and university: Why emotional intelligence matters. Gift. Educ. Int..

[96] Chan D.W. (2005). Self-perceived creativity, family hardiness, and emotional intelligence of Chinese gifted students in Hong Kong. J. Second. Gift. Educ..

[97] Peterson J.S., Pfeiffer S.I., Dedrick E.S., Foley-Nicpon M. (2018). Counseling gifted children teens. APA Handbook of Giftedness and Talent.

[98] Pfeiffer S.I., Stocking V.B. (2000). Vulnerabilities of academically gifted students. Spec. Serv. Sch..

[99] Silverman L.K., Kearney K. (1989). Parents of the extraordinarily gifted. Adv. Dev..

[100] Hackney H. (1981). The gifted child, the family, and the school. Gift. Child Q..

[101] Worrell F.C., Olszewski-Kubilius P., Subotnik R.F. (2012). Important issues, some rhetoric, and a few straw men: A response to comments on “rethinking giftedness and gifted education”. Gift. Child Q..

[102] Türkman B. (2020). The evolution of the term of giftedness & theories to explain gifted characteristics. J. Gift. Educ. Creat..

[103] Besnoy K.D., Swoszowski N.C., Newman J.L., Floyd A., Jones P., Byrne C. (2015). The advocacy experiences of parents of elementary age, twice-exceptional children. Gift. Child Q..

